# Osmotin: a plant sentinel and a possible agonist of mammalian adiponectin

**DOI:** 10.3389/fpls.2015.00163

**Published:** 2015-03-16

**Authors:** S. Anil Kumar, P. Hima Kumari, G. Shravan Kumar, C. Mohanalatha, P. B. Kavi Kishor

**Affiliations:** ^1^Department of Genetics, Osmania University, HyderabadIndia; ^2^Bioclues Organization, HyderabadIndia

**Keywords:** abiotic stress, adiponectin, biotic stress, OLPs, osmotin, protein–protein interactions

## Abstract

Osmotin is a stress responsive antifungal protein belonging to the pathogenesis-related (PR)-5 family that confers tolerance to both biotic and abiotic stresses in plants. Protective efforts of osmotin in plants range from high temperature to cold and salt to drought. It lyses the plasma membrane of the pathogens. It is widely distributed in fruits and vegetables. It is a differentially expressed and developmentally regulated protein that protects the cells from osmotic stress and invading pathogens as well, by structural or metabolic alterations. During stress conditions, osmotin helps in the accumulation of the osmolyte proline, which quenches reactive oxygen species and free radicals. Osmotin expression results in the accumulation of storage reserves and increases the shelf-life of fruits. It binds to a seven-transmembrane-domain receptor-like protein and induces programmed cell death in *Saccharomyces cerevisiae* through RAS2/cAMP signaling pathway. Adiponectin, produced in adipose tissues of mammals, is an insulin-sensitizing hormone. Strangely, osmotin acts like the mammalian hormone adiponectin in various *in vitro* and *in vivo* models. Adiponectin and osmotin, the two receptor binding proteins do not share sequence similarity at the amino acid level, but interestingly they have a similar structural and functional properties. In experimental mice, adiponectin inhibits endothelial cell proliferation and migration, primary tumor growth, and reduces atherosclerosis. This retrospective work examines the vital role of osmotin in plant defense and as a potential targeted therapeutic drug for humans.

## Introduction

Plants are subjected to various kinds of biotic (Lodge et al., 1993; Friedrich et al., 2000; Selitrennikoff, 2001; Kessler and Baldwin, 2002; Poupard et al., 2003; Anssour and Baldwin, 2010) and abiotic stresses (Brune et al., 1995; Apse and Blumwald, 2002; Rossel et al., 2002; Kaplan et al., 2004; Yamaguchi-Shinozaki and Shinozaki, 2006; Parent et al., 2008; Sanchez et al., 2008; Gill and Tuteja, 2010) during different developmental phases. These stresses impair many cellular activities, resulting in reduced growth and huge yield losses (Boyer, 1982; Wang et al., 2003; Rodriguez et al., 2005; Oerke, 2006). But, plants can sense and respond to these different stresses that are complex and integrative. Consequently, an array of cascade interactions evolve in the plants (Chisholm et al., 2006; Jones and Dangl, 2006; Atkinson and Urwin, 2012). Biotic stresses activate many intracellular defense signals leading to the production of antimicrobials and pathogenesis-related (PR) proteins (Vigers et al., 1991; Yun et al., 1997a; Veronese et al., 2003). PR proteins act as first line of plant defense and are induced in response to not only biotic but also to abiotic stresses (Bol et al., 1990; Linthotst and Van Loon, 1991; Stintzi et al., 1993; Van Loon, 1997). They were first observed in tobacco infected with tobacco mosaic virus and high levels of PR-5 proteins were detected in young leaves when exposed to salt stress (Van Loon and Kammen, 1970; Singh et al., 1989). Based on isoelectric point (pI), PR-5 proteins are divided into three groups: acidic (PR-S), basic (osmotin), and neutral (osmotin like proteins-OLPs; Koiwa et al., 1994; Van Loon and Van Strien, 1999). The counterparts of osmotin from tobacco PR-R and PR-S are acidic (Skriver and Mundy, 1990). But, *Glycine max* OLP (GmOLP) is an acidic protein (Onishi et al., 2006). PR-5 proteins are also called thaumatin-like proteins (TLPs) since they show structural homology with thaumatin, a protein isolated from *Thaumatococcus danielli* (Vander and Loeve, 1972; Edens et al., 1982; Cornelissen et al., 1986; Velazhahan et al., 1999). In spite of their high sequence similarity, even a small change in the amino acids of these proteins leads to diverse functions. Thaumatin tastes sweet but does not exhibit antifungal activity (Ogata et al., 1992; Zemanek and Wasserman, 1995). Contrarily, zeamatin does not taste sweet but exhibits high antifungal activity (Malehorn et al., 1994). Osmotin and OLPs accumulate in response to both biotic and abiotic stresses which facilitate the compartmentation of ions or solutes and exhibit antifungal activities. Adiponectin, the insulin sensitizing mammalian hormone is secreted in adipose tissues and exerts its function by binding to the plasma membrane receptors called as adiponectin receptors (AdipoRs). Adiponectin deficiency results in diabetes, fatty liver diseases, and cardiovascular disorders (Kadowaki and Yamauchi, 2005; Tang et al., 2005; Holland and Scherer, 2013). Adiponectin is a structural and functional homolog of osmotin (Narasimhan et al., 2005; Miele et al., 2011; Naseer et al., 2014). Husaini and Rafiqi (2012) and Viktorova et al. (2012) reviewed the importance of osmotin. But, the present paper deals with the review of progress made about the multiple activities of osmotin and identify the gaps in our understanding of osmotin protein, counterfeit for adiponectin.

## Expression of Osmotin during Various Stresses

Osmotin, a multifunctional stress responsive PR-5 protein is named on the basis of its induction by osmotic stress to low water potential (Singh et al., 1985). Osmotin and its homolog proteins are ubiquitous in most fruits and vegetables. Osmotin and OLPs confer stress tolerance to plants and their expression was induced by NaCl (LaRosa et al., 1987, 1989, 1992; Singh et al., 1987a; Bol et al., 1990; Raghothama et al., 1993, 1997; Zhu et al., 1993, 1995a; Koyama et al., 2001; Sokhansanj et al., 2006; Qureshi et al., 2007), abscisic acid (ABA; LaRosa et al., 1987, 1992; Singh et al., 1989; Raghothama et al., 1993, 1997; Zhu et al., 1993, 1995b), ethylene (LaRosa et al., 1992; Raghothama et al., 1993, 1997; Sato et al., 1996; Kitajima et al., 1998), dessication (Pla et al., 1998), cold (Newton and Duman, 2000; D’Angeli and Altamura, 2007), drought (Parkhi et al., 2009), salicyclic acid (Kim et al., 2002), wounding (LaRosa et al., 1992; Zhu et al., 1995a), bacterial (Choi et al., 2013), viral (Cornelissen et al., 1986; Stintzi et al., 1991; LaRosa et al., 1992; Elvira et al., 2008; Choi et al., 2013), and fungal stresses (Woloshuk et al., 1991; Vigers et al., 1992; Raghothama et al., 1993; Liu et al., 1994; Zhu et al., 1995a, 1996; Abad et al., 1996; Zuker et al., 2001; Tzou et al., 2011). However, the signaling pathways associated with the induction of osmotin by these different stresses are not known. Transgenic and native expression of osmotin and OLPs was observed in various plants when treated with different biotic and abiotic stresses (**Tables 1** and **2**). Osmotin is induced in *Petunia* when treated with *Penicillium funiculosum, Erwinia stewartii, Pseudomonas syringae*, aspirin, wounding, and salicylic acid (Kim et al., 2002). When treated with salt, *Osmotin*34 was induced in *Bruguiera gymnorhiza* transgenics expressing ankyrin repeat protein 1 (BgARP1; Miyama and Tada, 2011). It is also induced in genetic tumors of tobacco plants (Fujita et al., 1994) and at high atmospheric CO_2_ concentration in potatoes (Plessl et al., 2007) and in leaves and trichomes of tobacco by cadmium metal stress (Harada et al., 2010) implying that it plays a vital role in this diverse array of stresses. *Osmotin* gene transferred to somatic embryos of tea plants showed an increase in seed storage reserves and desiccation tolerance in recalcitrant embryos (Bhattacharya et al., 2006). Osmotin levels are reduced in virus induced gene silencing of *CaOXR1* (*Capsicum annum* oxidoreductase1) and *CaOXR1/CaRAV1* (*Capsicum annum* related to *ABI3/VP1*) in pepper leaves when treated with NaCl or mannitol (Lee et al., 2010b). Expression of *osmotin* in transgenics showed enhanced fruit shelf-life in strawberries (Chen, 2012). It is also induced with fungicide acibenzolar-*S*-methyl treatment which may act as an elicitor (Whan et al., 2009). Thus, it appears *osmotin* is expressed differentially by many stresses and has multifaceted roles to perform in plants.

**Table 1:** Transgenics developed using osmotin and osmotin like proteins (OLPs).

Gene	Isolated from	Validated in	Phenotypic effects of transgenic plants	Reference
*Osmotin*	Tobacco	Potato	Tolerance against *Phytophthora infestans*	Liu et al. (1994)
*Osmotin* and truncated *Osmotin*	–	Tobacco, potato	Resistance to *Phytophthora infestans*	Liu et al. (1996)
OLP	–	Potato	–	Zhu et al. (1996)
OLP	–	Tobacco	Ethylene responsive elements and ERF3	Kitajima et al. (1998)
OLP	–	Potato	Tolerance to salt	Evers et al. (1999)
*Osmotin*	Tobacco	Potato	Tolerance against *Phytophthora infestans*	Li et al. (1999)
*Osmotin*	Tobacco	Peppermint	–	Niu et al. (2000)
*Osmotin*	Tobacco	Tobacco	Tolerance to osmotic stress	Barthakur et al. (2001)
*Osmotin*	*Nicotiana tabacum*	*Dianthus caryophyllus*	*Fusarium oxysporum*	Zuker et al. (2001)
*Osmotin*	Tobacco	Tomato	Enhanced tolerance to cold stress	Sarad et al. (2004)
*Osmotin*	–	*Brassica juncea*	Increased resistance to *Alternaria brassicae*	Taj et al. (2004)
*Osmotin*		Tobacco	Enhanced salt stress	Zhang et al. (2004)
*Osmotin*	Tobacco	Tomato	Resistance to *Fusarium* wilt	Ouyang et al. (2005)
*Osmotin*	Tobacco	Tea	Tolerance to dessication and accumulation of storage reserves	Bhattacharya et al. (2006)
*Osmotin*	–	Tobacco	Tolerance to salt	Sokhansanj et al. (2006)
*Osmotin*		Tobacco	Resistance to *Pseudomonas syringae*	Qin et al. (2006)
*Osmotin*	Tobacco	*Olea europaea*	Tolerance to cold	D’Angeli and Altamura (2007)
*Osmotin*	Tobacco	Strawberry	Enhanced tolerance to salt	Husaini and Abdin (2008)
*Osmotin*	–	Wheat	Increased root growth	Noori and Sokhansanj (2008)
*Osmotin*	–	*Tecomella undulata*	–	Aslam et al. (2009)
*Osmotin*	Tobacco	Cotton	Increased tolerance to drought	Parkhi et al. (2009)
*Osmotin*	Tobacco	Tomato	–	Randhawa et al. (2009)
*Osmotin*	Tobacco	Tomato	Tolerance to salt and drought	Goel et al. (2010)
*Osmotin*	Tobacco	Mulberry	Tolerance against biotic and abiotic stresses	Das et al. (2011)
*Osmotin*	Tobacco	*Medicago sativa*	–	Kancharla (2011)
*Osmotin*	Tobacco	Rice	–	Rao et al. (2011)
*Osmotin*	Tobacco	Rice	–	Sripriya et al. (2011)
*Osmotin*	Tobacco	Chili pepper	Enhanced salt tolerance	Subramanyam et al. (2011)
Recombinant *Osmotin* (*rOSM*)	Tobacco	*E. Coli*	Resistance against *Cryptococcus neoformans, Candida albicans, Saccharomyces cerevisiae* and *Pichia methanolica*	Tzou et al. (2011)
Recombinant Tobacco *Osmotin*	Tobacco	Strawberry	Tolerance to* Aspergillus niger*	Chen (2012)
*Osmotin*	Tobacco	Strawberry	Tolerance to *Piriformospora indica*	Husaini et al. (2012)
AP24 osmotine	Potato	Tobacco	Resistance to *P. infestans* and *R. solani*	Rivero et al. (2012)
*Osmotin* (*Tbosm*)	Tobacco	soybean	Resistance to salinity stress and fungal infections	Subramanyam et al. (2012)
OLP	*Solanum nigrum*	Peanut	Enhanced disease resistance to late leaf spot	Vasavirama and Kirti (2012)
OLP (CaOSM1)	*Capsicum annuum*	*Arabidopsis*	Increased tolerance to *Pseudomonas syringe pv. tomato* and *Hyaloperonospora arabidopsidis*	Choi et al. (2013)

**Table 2:** Stress response of native plants overexpressing osmotin and OLPs.

Osmotin/OLP	Native expression	Expressed during	Reference
Osmotin	Tobacco	Salt stress	LaRosa et al. (1989)
Osmotin	Tobacco	Treated with auxin	Grosset et al. (1990)
Osmotin	Tobacco	Viral infection and wounding	Neale et al. (1990)
OLP	Tobacco	Salt stress	Takeda et al. (1991)
OLP (pA8 and pA9)	*Atriplex nummularia*	Salt adapted and undapted cells	Casas et al. (1992)
Osmotin	Tobacco	Salt stress	Kumar and Spencer (1992)
Osmotin	Tobacco	Water deficit and ABA stress	LaRosa et al. (1992)
Osmotin	Tobacco	Treatment with ABA	Nelson et al. (1992)
OLP	*Mesembryanthemum crystallinum*	Treatment with salt and cytokinin	Thomas and Bohnert (1993)
Osmotin	Tobacco	Ethylene/Methyl jasmonate	Xu et al. (1994)
OLP	Tobacco	Treatment with ethylene	Sato et al. (1996)
OLP	Potato	Treatment with *Phytophthora infestans*	Takemoto et al. (1997)
OLP (QsOLP)	*Quercus suber*	Oxidative stress	Pla et al. (1998)
Osmotin	Tomato	*Pseudomonas syringae*	Jia and Martin (1999)
OLP	*Chicorium*	Emryonic cell cultures	Helleboid et al. (2000)
Osmotin	Pepper	*Xanthomonas campestris pv. Vesicatoria* infection	Jung and Hwang (2000)
OLP	*Solanum dulcamara*	Cold stress	Newton and Duman (2000)
OLP	*Benincasa hispida*	–	Shih et al. (2001)
Osmotin (PhOSM)	*Petunia hybrida*	*Penicillium funiculosum, Erwinia stewartii, Pseudomonas syringae*, aspirin, salicylic acid and wounding	Kim et al. (2002)
Osmotin	Tobacco	Water deficit	Kawaguchi et al. (2003)
Osmotin	Tobacco	With/without Ethylene	Lee and Kim (2003)
OLP	*V. vinifera* L.	environmental and/or pathological	Monteiro et al. (2003a)
Osmotin	*V. vinifera L.*	*Phomopsis viticola, Botrytis cinereamycelia and U. necator*	Monteiro et al. (2003b)
Osmotin	*V. vinifera L.*	Salt stress	Agaoglu et al. (2004)
OLP (CAOSM1)	*Capsicum annuum* L.	Infection with *X. campestris* pv. *Vesicatoria, Colletotrichum coccodes, Phytophthora capsici*, ethylene, methyl jasmonate, high salinity, cold acclimation and mechanical wounding	Hong et al. (2004)
OLP	Sugar beet	Osmotic stress tolerance	Hajheidari et al. (2005)
Osmotin	*Capsicum annuum*	Infection with *X. campestris* pv. *vesicatoria*	Lee and Hwang (2005)
Osmotin	Cotton	Treatment with ethephon and hydrogen peroxide (H_2_O_2_)	Wilkinson et al. (2005)
OLP	Chardonnay wine	–	Okuda et al. (2006)
OLP (GmOLPa)	*Glycine max*	Salt and dehydration stress	Onishi et al. (2006)
Osmotin	Rice	Salt stress	Tanaka et al. (2006)
OLP (SniOLP)	*Solanum nigrum*	Inhibition of *Rhizoctonia batiticola* and *Sclerotinia sclerotiorum*	Jami et al. (2007)
Osmotin	Tobacco	Osmotic stress tolerance	Qureshi et al. (2007)
OLP (FaOLP2)	Strawberry	Salicylic acid, abscisic acid (ABA), or mechanical wounding	Zhang and Shih (2007)
OLP	Potato	Osmotic stress tolerance	Aghaei et al. (2008)
OLP	*Capsicum chinense*	Infected with pepper mild mottle virus	Elvira et al. (2008)
Osmotin	Rice	Cold and salt stress	Huang et al. (2008)
Osmotin	Mandarin	Drought stress	Gimeno et al. (2009)
OLP (GmOLPa and GmOLPb)	*G. max*	Salt, methyl jasmonate and salicylic acid	Tachi et al. (2009)
Osmotin	*Bruguiera gymnorhiza*	Salt stress	Tada and Kashimura (2009)
Osmotin	*Brassica napus*	*Sclerotinia sclerotiorum*	Zhao et al. (2009)
Osmotin	*P. euphratica*	Salt stress	Brinker et al. (2010)
OLP	Potato	Induced when infected *with P. infestans*	El-Komy et al. (2010)
Osmotin	Tobacco	Cd stress	Harada et al. (2010)
Osmotin	*Capsicum annuum*	Osmotin levels are suppressed in leaves of virus induced gene silencing of *CaOXR1 and CaOXR1/CaRAV1* upon treatment with NaCl or mannitol	Lee et al. (2010b)
Osmotin	*Arabidopsis*	Infection with *Alternaria brassicicola*	Mukherjee et al. (2010)
			
Osmotin (CpOsm)	*Calotropis procera*	Antifungal activity against *F. solani, Neurospora* sp., *Colletotrichum gloeosporioides*	de Freitas et al. (2011a)
Osmotin (CpOsm)	*Calotropis procera*	Inhibits *F. solani* spore and hyphae	de Freitas et al. (2011b)
OLP	*Vitis vinifera*	Infected with Flavescence dore’e	Margaria and Palmano (2011)
Osmotin	Tobacco	–	Miele et al. (2011)
Osmotin	*Anthemis nobilis*	Salinity stresses and iron deficiency	Siahsar et al. (2011)
Osmotin	*Calotropis*	*F. solani, Neurospora sp.* and *Colletotrichum gloeosporioides, F. oxysporum, R. solani, A. niger*	Souza et al. (2011)
Osmotin	Tomato	Addition of nitrogen	Fatima et al. (2012)
PcOSM1and PcOSM2	*Piper colubrinum*	*Phytophthora capsici* and *F. oxysporum*	Mani et al. (2012)
Osmotin	*Capsicum annuum* L.	Cold stress	Patade et al. (2012)
Osmotin	Tobacco	Salt adapted	Trivedi et al. (2012a)
Osmotin	*Olea europaea*	Cold stress	D’Angeli et al. (2013)
Osmotin	*Arabidopsis*	Low water potential	Sharma et al. (2013b)

## Differential and Developmental Regulation of Osmotin

Osmotin and OLPs show spatial and temporal regulation during various stages of development in roots, pollen, pistils, and fruits (Kononowicz et al., 1992; Kim et al., 2002). While osmotin is secreted in an intracellular compartment, its counterpart lacking 20 C-terminal amino acids is secreted into extracellular matrix (Liu et al., 1996; Parkhi et al., 2009). The lack of the C-terminal vacuolar sorting motif in GmOLPa results in secretion of GmOLPa into extracellular space instead of the vacuole while GmOLPb with C-terminal elongation is secreted into the vacuole (Onishi et al., 2006; Tachi et al., 2009).

Osmotin shows tissue-specific activity with ABA, ethylene, and NaCl treatments (Raghothama et al., 1997). Osmotin expression was observed in flowers of tobacco (Neale et al., 1990), somatic embryos (Bhattacharya et al., 2006), leaves, and trichomes (Harada et al., 2010), skin of grapes (Margaria and Palmano, 2011), seed coat and endosperm in olive (D’Angeli et al., 2013).

Osmotin like proteinss also show tissue-specific expression in many plants. Under oxidative stress conditions, OLPs are expressed in the quiescent region of root apex and meristematic region of shoot apex (Pla et al., 1998). Activity of OLPs was also observed in flowers and fruits of tomato (Chen et al., 1996), roots, stems, leaves, and flowers of *Arabidopsis* (Capelli et al., 1997), root and shoot apices of *Quercus suber* (Pla et al., 1998), ovary of grapes (Salzman et al., 1998), root, stem, leaves, and flowers of *Solanum nigrum* (Jami et al., 2007).

Abscisic acid transcriptionally regulates *osmotin* and *osmotin* promoter in tobacco (LaRosa et al., 1992; Nelson et al., 1992). In ABA-deficient mutants of tomato, *osmotin* transcripts are not induced by salt and water stresses suggesting the endogenous levels of ABA are required for the transcript accumulation (Grillo et al., 1995). Along with *cis*-elements, ethylene-responsive element binding proteins (EREBPs) which bind to two GCC boxes in osmotin promoter are essential for ethylene response (Raghothama et al., 1997; Xu et al., 1998). *Osmotin* expression was influenced by the type of promoter used. *Osmotin-*specific promoter showed higher expression levels compared to *CaMV35S* promoter in sugar beet cells (Ivic-Haymes and Smigocki, 2005). Osmotin promoter is also induced by wounding in sugar beet and apple (Snyder et al., 1999; Liu et al., 2001). OPBP1 (*osmotin* promoter binding protein 1), an apetala2/ethylene responsive transcription factor of tobacco displayed salt and disease tolerance in rice (Chen and Guo, 2008). Jasmonate Ethylene Response Factor 1 (JERF1) and GbERF (*Gossypium barbadense* Ethylene Response Factor) induces *osmotin* expression by activating the GCC box (Zhang et al., 2004; Qin et al., 2006). Constitutive expression of OLPs was observed due to ethylene responsive elements (AGCCGCC) and ethylene-responsive element binding factor (ERF3) in cultured tobacco cells (Sato et al., 1996; Kitajima et al., 1998), and transcriptional activation of OLP (OSMLl3 and OSML8l) promoters was also studied in potato (Zhu et al., 1995a). Thus, the information is fragmentary and only a few of the plant hormones mentioned above regulate osmotin and OLPs, but we still do not know the comprehensive regulation of osmotin at the transcriptional or post-transcriptional level by other phytohormones.

## Characterization and Structure of Osmotin

Singh et al. (1987a) characterized tobacco osmotin protein. It is a 26-kDa cationic protein induced in cultured cells of tobacco adapted to NaCl and low water potential, and accumulates up to 12% of total cell protein. Without salt exposure also, osmotin is accumulated in untreated cells and NaCl-treated cell cultures (Singh et al., 1985, 1987b; Bressan et al., 1987). This indicates that basal levels of osmotin may perhaps be essential for housekeeping in the cells that are not exposed to stress. Osmotin occurs in two forms as osmotin I and II in 2:3 ratios with basic pIs of about 8.2. Osmotin I (aqueous soluble form) and osmotin II (detergent soluble form) have the same first 22 amino acids at N-terminus. But, osmotin II is more resistant than osmotin I to protease digestion and cross reacts with antibodies of osmotin I. Anti-osmotin antibodies cross react with *Arabidopsis* TLP (ATLP-1) protein also (Hu and Reddy, 1997). Osmotin shows significant sequence homology at amino acid level with the sweet-tasting protein thaumatin and shares several similar characteristic features like molecular weight, lack of sulfhydral residues, basic pI, disulfide bonds, and high proline content. Osmotin is not sweet in taste and the probable reason may be due to lack of lysine residues that are present in thaumatin protein (Richardson et al., 1987). Osmotin contains 16 cysteine residues resulting in the formation of eight disulfide linkages; alanine is present at the cleavage site of the N-terminal leader sequence (Kononowicz et al., 1994). It may also act as a storage protein which is evident by its accumulation in vacuolar inclusion bodies, with the help of C terminal peptide (Melchers et al., 1993). Osmotin activity was completely reduced with increasing concentrations of K^+^ but, the same was retained even at high concentrations of Ca^2+^, suggesting that Ca^2+^ facilitates osmotin binding to the fungal cell surface (Salzman et al., 2004). Interestingly, Izh2p a protein from *Saccharomyces cerevisiae* also serves as a receptor for osmotin (Kupchak et al., 2008).

Min et al. (2004) analyzed the crystal structure of osmotin. Purified osmotin from salt-adapted cells was used for structural analysis at 2.3 Å resolution and compared with other PR-5 proteins. Osmotin shows two monomers with slight difference in the tertiary structure and presence of a non-crystallographic dimer in the asymmetric unit. Osmotin is composed of three domains and the folds are very much similar to thaumatin, zeamatin, and tobacco PR-5d protein (de Vos et al., 1985; Batalia et al., 1996; Koiwa et al., 1999). Osmotin shows a prominent cleft assembled by domains I and II. Domain I consists of 11 β strands which form the core of the protein, while several loops of domain II extended from domain I are stabilized by four disulfide bonds, and domain III shows a small loop with two disulfide bonds. The acidic cleft of domain I and II is formed by acidic residues Glu84, Asp97, Asp102, and Asp185. But, osmotin lacks thaumatin loop which is responsible for its sweetness in domain II (Slootstra et al., 1995). Homology modeling of *Piper colubrinum* osmotin2 (PcOSM2) showed domain I with 12-β sheets, an α-helical domain II, and domain III with 2-β sheets, contrarily PcOSM1 exhibited a deformed, unidentical domain III and loss of 4-β sheets in domain I. But, the acidic cleft responsible for antifungal activity was present in both the isoforms (Mani et al., 2012).

## Molecular Mechanism of Osmotin Action

The mode of action of osmotin is not well understood, however, osmotin protects the cells from osmotic shock especially during abiotic stresses by compartmentalization of solutes or by structural or metabolic alterations (Singh et al., 1987a; Barthakur et al., 2001). Besides osmotic balance, it plays a crucial role as an antifungal protein. Several hypotheses were proposed to explain the molecular action of osmotin. Firstly, osmotin with the help of cell wall components is involved in permeabilization of membrane and plasmolysed cells become insensitive to osmotin suggesting that the cell wall components are needed for its activity (Abad et al., 1996). In the second mechanism, osmotin subverts cell signal transduction pathway in the target by activating mating pheromones to weaken the cell wall and increases its cytotoxic efficacy (Yun et al., 1998). The third proposed mechanism for antifungal activity is the interaction of osmotin protein with the receptor of cell membrane which initiates mitogen activated protein kinase signal transduction pathway, leading to the formation of a transmembrane pore to cause leakage in membrane and subsequent rupture of the membrane (Bowles, 1990; Roberts and Selitrennikoff, 1990; Cheong et al., 1997; Yun et al., 1997b, 1998; Anzlovar et al., 1998; Narasimhan et al., 2001, 2005). In general, PR-5 proteins function similar to β-1,3-glucanase by hydrolyzing the β-1,3-glucans of the pathogens (Grenier et al., 1999). Osmotin is also involved in apoptosis through the accumulation of reactive oxygen species (ROS) *via* the RAS2/cAMP pathway as shown in *Saccharomyces cerevisiae* (Narasimhan et al., 2001).

## Osmotin Mediates Signal Transduction and Programmed Cell Death

Plants perceive external signals from environment and manifest mechanisms to acquire stress tolerance through multiple signal transduction pathways. Osmotin (PhOSM) is involved in signal transduction during wound stress (Kim et al., 2002). The mitogen-activated protein kinase (MAPK) cascade is a critical signaling pathway associated in response to external stimuli and contains three consecutively activated kinases. Active MAPKKK (mitogen-activated protein kinase kinase kinase) activates MAPKK (mitogen-activated protein kinase kinase) by phosphorylation, which in turn phosphorylates MAPK. The activated MAPK phosphorylates target proteins and regulates the *osmotin* gene. MAPKs are activated when exposed to stress conditions like salt, drought, cold, and pathogen attack (Jonak et al., 1996; Mikolajczyk et al., 2000). Overexpression of *Gossypium hirsutum* MPK2 (GhMPK2), a MAPK in tobacco showed upregulation of osmotin with enhanced tolerance to salt and drought, which suggests that GhMPK2 has a role in signal transduction (Zhang et al., 2011). Based on a bioinformatics study, osmotin confers tolerance against biotic and abiotic stress through its involvement in signal transduction pathway, and not activating a transcription factor. When analyzed with bioinformatics tools, DNA binding motif was not found in osmotin and it has only 0–20% homology with protein sequences from database of *Arabidopsis* transcription factors which confirms that it has no DNA binding motif. Further, superimposition of 3D-modeled structure of osmotin with *Arabidopsis* transcription factors also suggests the absence of DNA-binding motifs (Abdin et al., 2011).

Hypersensitive reaction (HR) is a consequence of disease resistance in plants and cell death occurs either by apoptosis or programmed cell death (PCD). PCD plays a crucial role in plant development and host interaction, which is activated by cell wall components or toxins or proteins secreted from pathogens (Aliprantis et al., 1999). P^53^ is overexpressed in cells treated with toxins and the balance is influenced by hormone or toxin. P^53^ acts as a link between cell cycle and PCD. Osmotin decreases the pathogenesis by interacting with the cell cycle machinery and overexpresses the cell cycle components which inhibit the cell death pathway components like P^53^. *Brassica juncea* calli overexpressing osmotin showed delayed symptoms when treated with *Alternaria* toxin, suppressed the expression of P^53^ and the activity of caspase I was not affected which shows that osmotin is involved in P^53^-mediated PCD pathway (Taj et al., 2004). PCD was observed in *Saccharomyces cerevisiae* BWG7a cells when treated with different concentrations of osmotin due to suppression of transcription of the stress responsive genes with the accumulation of ROS (Narasimhan et al., 2001).

## Role of Osmotin during Salt Stress Tolerance

Osmotin plays an important role in salt stress tolerance by sequestering Na^+^ ions and compartmentalizing them into vacuoles and intercellular spaces. The association of tobacco osmotin protein with tonoplast (Singh et al., 1987a) and the OLP identified from *Mesembryanthemum crystallinum* suggests the role of osmotin in the intracellular compartmentation of Na^+^ ions (Yen et al., 1994). But, whether osmotin upregulates sodium-proton antiporter1 (*NHX1*) gene to sequester Na^+^ ions or how it is able to perform this functions is not yet clear. It is also not clear if osmotin has a direct role to play or it stimulates other proteins that are downstream. Transgenics overexpressing *osmotin* gene exhibited salt tolerance in potato (Evers et al., 1999), tobacco (Barthakur et al., 2001), *Triticum aestivum* cv. Marvdasht (Noori and Sokhansanj, 2008), strawberry (Husaini and Abdin, 2008), tomatoes (Goel et al., 2010), mulberry (Das et al., 2011), chili pepper (Subramanyam et al., 2011), and soybean (Subramanyam et al., 2012) by retaining chlorophyll, preventing the accumulation of ROS, with an increase in relative water content, proline accumulation, increase in root length, shoot length, plant height, leaf expansion, and improved root growth than controls. Overexpression of OLP lacking short C terminal cDNA also showed such an enhanced salt tolerance in potato (Evers et al., 1999). Transgenic mulberry expressing *osmotin* driven by *CaMV35S* promoter displayed better tolerance to salt stress than the transgenics containing* osmotin* under the influence of* rd29A* promoter, though the *rd29A* promoter is responsive to dehydration while the *CaMV35S* promoter is constitutive (Das et al., 2011). Rice transgenic plants expressing OPBP1 showed salt tolerance with enhanced root length and root growth than the untransformed controls (Chen and Guo, 2008). This suggests that osmotin somehow triggers auxin biosynthesis and improves root biomass under salt stress.

## Role of Osmotin in Drought and Cold Tolerance

Osmotin and OLPs also accumulate during drought stress. Accumulation of osmotin mRNA and osmotin protein varies in different tissues with different treatments and *vice-versa*. Osmotin mRNA was observed in different plant tissues when stimulated with water deficit and ABA, whereas osmotin protein was not detected. During water deficit conditions, the number of ribosomes loaded was not affected in apical leaves but, a higher level of messenger RNA was noticed in basal leaves (LaRosa et al., 1992; Kawaguchi et al., 2003). Transgenics expressing *osmotin* showed an increase in the relative water content, chlorophyll, and leaf expansion than controls and recovered completely after rewatering. This implies that osmotin is able to protect chlorophyll and photosynthetic machinery under water limited conditions. Transgenics are tolerant to water deficit conditions in tobacco (Barthakur et al., 2001) and tomato (Goel et al., 2010). Transgenic mulberry plants expressing *osmotin* with rd29A promoter are more responsive to drought than mulberry plants expressing *osmotin* with *CaMV35S* promoter (Das et al., 2011). Thus, it appears that stress-inducible promoters are better for the overexpression of *osmotin* gene compared to universal promoters.

Plants are very sensitive to cold stress and results in depolarization and rigidification of cell membrane when exposed to low temperatures (Los and Murata, 2004). Pollen sterility occurs if the plants are exposed to <20^∘^C for few days at young microspore stage in rice. During the cold conditions, the tapetal cell which nourishes the pollen undergoes hypertrophy leading to the formation of sterile pollen grains with little or no starch and it was observed that anthers show more abnormalities than pistils or other floral organs in cold exposed rice plants (Imin et al., 2006). Osmotin also plays a role in cryoprotection during low temperature exposure. During cold stress, osmotin is induced in seed coat and endosperm in olive, and OLP in pollen of *Solanum* (Volger and Heber, 1975; Zhu et al., 1993; D’Angeli et al., 2013). Increased osmotin promoter activity was also observed in tobacco pollen grains under normal conditions (Kononowicz et al., 1992). Osmotin protein homolog was upregulated when plants are exposed to 12^∘^C in the cold sensitive rice cultivar Doongara (Imin et al., 2006). D’Angeli and Altamura (2007) demonstrated that osmotin is involved in PCD, which is cold inducible in olive trees. The results suggest that osmotin regulates cytoskeleton alterations and mediates calcium signaling under cold stress. A change in Ca^2+^ concentration may initiate the cold induced PCD (Kratsch and Wise, 2000) which has been elucidated in human cells (Risso et al., 1998) and yeast (Narasimhan et al., 2001, 2005). Overexpression of osmotin enhanced the tolerance to cold in tomato growing at higher altitudes (Sarad et al., 2004). *Capsicum*, upon treatment with cold stress showed enhanced *osmotin* transcripts (Patade et al., 2012). OLP was isolated from frozen-thawed protoplasts with a molecular mass of 25 kDa in *Solanum dulcamara* (Newton and Duman, 2000). Thus, these findings implicate osmotin during cold stress tolerance. However, it is unknown if it induces the biosynthesis of fatty acids that are associated with cell membrane protection under cold stress, conditions or upregulates cold-regulated genes.

## Role of Osmotin in Proline Accumulation

Proline, a multifunctional molecule, accumulates during stress and accounts for up to 80% of the total amino acid pool in certain plants. It acts as an osmotic agent and as free radical scavenger that helps plants to withstand drought and salt stress conditions (Kishor and Sreenivasulu, 2014). Proline, when accumulated in cytosol, does not show any detrimental effects but detoxifies ROS and free radicals by forming long living adducts during osmotic stress (Floyd and Nagy, 1984; Lutts et al., 1996; Hong et al., 2000; Vinocur and Altman, 2005). Osmotin triggers the accumulation of osmolytes like proline and glycine betaine (Holmstrom et al., 2000) and the accumulation of proline in *osmotin* overexpressed transgenics is influenced by both constitutive and inducible promoters. While higher proline accumulation was reported in transgenics overexpressing *osmotin* such as potato (Evers et al., 1999), tobacco (Barthakur et al., 2001; Sokhansanj et al., 2006), tomato (Goel et al., 2010), mulberry (Das et al., 2011), and chili pepper (Subramanyam et al., 2011), transgenics without proline accumulation were also noticed but with relatively low stress tolerance (Nanjo et al., 1999). Thus, osmotin expression in transgenics confers osmotic tolerance by accumulation of more proline. Nevertheless, it is necessary to find out if there is any upregulation of *P5CS* or *P5CR* genes involved in proline biosynthetic pathway by *osmotin* overexpression.

## Role of Osmotin in Antioxidant Defense

While the production of ROS increases, the antioxidative system is impaired under many environmental stress conditions (Dhindsa and Matowe, 1981). It has been observed that accumulation of hydrogen peroxide (H_2_O_2_) was lower in transgenics overexpressing *osmotin* than the corresponding controls when treated with different NaCl concentrations implying that osmotin helps in controlling its overaccumulation. Transgenic *Capsicum annum* L. overexpressing osmotin when treated with salt, showed higher activity of ascorbate peroxidase (APX) and superoxide dismutase (SOD) to detoxify the accumulated H_2_O_2_ than the untransformed controls under identical conditions. But, how osmotin activates the APX and SOD activities is not clear. Low levels of malondialdehyde, an indicator of lipid peroxidation levels in transgenics overexpressing *osmotin* compared to controls, suggests less damage of cell membrane in transformants compared to controls (Subramanyam et al., 2011). Thus, *osmotin* overexpressing plants neutralize the ROS by producing more compatible solutes or expression of specific antioxidative enzymes.

## Antifungal Activity of Osmotin

Fungus results in massive crop losses, crop rotations, and fungicides are not fully effective in controlling fungi (Selitrennikoff, 2001). PR proteins are effective in controlling pathogens and *osmotin* has specific and broad spectrum activity (Abad et al., 1996; Veronese et al., 2003). Osmotin acts as an antifungal cytotoxic compound with rapid cell death in the yeast (Kupchak et al., 2008). Cell wall components especially oligosaccharides act as elicitors of plant defense (Ebel, 1998). Osmotin requires cell wall components for its action and plasmolysed *Trichoderma longibrachiatum* fungal cells are resistant to osmotin action (Abad et al., 1996). However, osmotin activity varies with change in the cell wall composition (Ibeas et al., 2000, 2001). The outer layer of the yeast cell wall is composed of mannoproteins, a surface determinant for osmotin. Phosphomannan is an essential polyanion for osmotin binding to the cell wall. Phosphomannoproteins are regulated by MNN1, MNN2, MNN4, or MNN6 which facilitate the binding of osmotin to the cell wall and are responsible for cytotoxicity. MNN1 adds terminal mannose making osmotin unable to bind to cell wall and null *mnn1* mutants exhibit enhanced osmotin binding and sensitivity. Salt and carbohydrate disturb the interaction between phosphomannan and osmotin. Overexpression of cell wall protein containing inverted repeats (PIR proteins) results in enhanced resistance to osmotin and deletion results in sensitivity to osmotin in resistant strains (Yun et al., 1997b). *Fusarium oxysporum* f. sp. *Nicotianae* overexpressing cell wall glycoprotein PIR2 exhibits increased resistance to osmotin (Narasimhan et al., 2003). Cell walls of pathogens possess proteins that expedite or restrict plant defense proteins to act on the plasma membrane. Specificity exists between osmotin and its target cell. Strains sensitive to tobacco osmotin are resistant to *Atriplex nummularia* OLP and all spheroplasts of resistant and susceptible strains are equally sensitive to the toxicity of osmotin and not to OLPs. Osmotin binding to the cell wall in *Aspergillus nidulans* is mediated by a heteromeric G-protein through a signal transduction pathway and its binding is inhibited by guanosine 5′-O-(2-thiodiphosphate) βS (GDPβS) that blocks G-protein. Mutation in *FadA* (α-subunit) and deletion in *SfaD* (β subunit) of G-protein blocks the osmotin binding and displays increased resistance to osmotin. These mutants showed enhanced chitin content with decreased sensitivity to osmotin (Coca et al., 2000). *Sachharomyces cerevisiae* D1 (SSD1) regulates the deposition of glucans and PIR glycoproteins in the cell wall, a major determinant of osmotin resistance in yeast and susceptibility to osmotin in *Saccharomyces cerevisiae* is encoded by *Fusarium* osmotin resistance3 (FOR3), a homolog of SSD1. *Δfor3* mutants showed high sensitivity to osmotin similar to *Δssd1* mutants (Lee et al., 2010a). It was discovered that PH036, an osmotin binding plasma membrane protein is required for full sensitivity of osmotin (Narasimhan et al., 2005).

Osmotin transcript levels vary during growth and decrease with advancing maturity in grapevine berries (Kretschmer et al., 2007). Osmotin transcript accumulation was reported during incompatible plant pathogen interaction with tomato Pto and *Pseudomonas syringae* pv. Avr Pto genes (Jia and Martin, 1999). *In vitro* analysis demonstrates that osmotin is effective against different fungal pathogens (Yun et al., 1997a). Osmotin and OLPs are expressed during infection and confer antifungal activity against a broad range of fungal species like *Phytophthora infestans* (Woloshuk et al., 1991; Vigers et al., 1992; Zhu et al., 1993, 1995b; Liu et al., 1994, 1996; Plessl et al., 2007; Rivero et al., 2012), *Candida albicans*, *Neurospora crassa*, and *Trichoderma reesei* (Vigers et al., 1992), *Guignardia bidwellii* and *Botrytis cinerea* (Salzman et al., 1998), *Phomopsis viticola*, *Botrytis cinereamycelia*,* and Uncinula necator* (Monteiro et al., 2003b), *Xanthomonas campestris* pv. *Vesicatoria*, *Colletotrichum coccodes*, and *Phytophthora capsici* (Hong et al., 2004), *X. campestris* pv. *Vesicatoria* (Lee and Hwang, 2005), *F. oxysporum* f. sp. *Lycopersicii* (Ouyang et al., 2005), *Rhizoctonia batiticola* and *Sclerotinia sclerotiorum* (Jami et al., 2007), pepper mild mottle virus (Elvira et al., 2008), *Sclerotinia sclerotiorum* (Zhao et al., 2009*), Alternaria brassicicola* (Mukherjee et al., 2010), *F. solani*, *Neurospora* sp., and *Colletotrichum gloeosporioides*, *F*. *oxysporum*, *R. solani*, *Aspergillus niger* (de Freitas et al., 2011a,b; Souza et al., 2011; Rivero et al., 2012), *Piriformospora indica* (Husaini et al., 2012), *Phytophthora capsici* and *F. oxysporum* (Mani et al., 2012), *Microsphaera diffusa*, *Septoria glycines* and *Phakopsora pachyrhizi* (Subramanyam et al., 2012) and *Phaeoisariopsis personata* (Vasavirama and Kirti, 2012). Compared to controls, enhanced activity of osmotin was observed in transgenic mulberry plants and in particular, transgenics expressing osmotin with *rd29A* promoter showed more tolerance to *F. pallidoroseum*, *Colletotrichum dematium*, and *Colletotrichum gloeosporioides* than transgenics with *CaMV35S* promoter (Das et al., 2011). Transgenic rice overexpressing *OPBP1* exhibited increased disease resistance against *Magnaporthe oryzae* and *R. solani* (Chen and Guo, 2008). OLPs are also involved in plant defense and translocate along with actin filaments during cytoplasmic aggregation (Takemoto et al., 1997).

## Role of Adiponectin and Its Receptors in Mammals

Adiponectin, a mammalian circulating protein produced in adipose tissue is an insulin-sensitizing hormone (Turer and Scherer, 2012). Adiponectin is also referred as Acrp30, AdipoQ, apM1, and GBP28 (Scherer et al., 1995; Hu et al., 1996; Maeda et al., 1996; Nakano et al., 1996). Adiponectin occurs in plasma as high, medium, and low molecular weight forms (HMW, MMW, and LMW). Many studies suggest that the HMW form is closely associated with insulin sensitivity (Bobbert et al., 2005; Salani et al., 2006). Hence, we have considered HMW adiponectin in the present study. It exerts its functions by binding to the plasma membrane receptors called AdipoRs. Adiponectin and its receptors are well reviewed by Kadowaki and Yamauchi (2005). AdipoRs are of two types: AdipoR1 that activates the 5′ adenosine monophosphate-activated protein kinase (AMPK) pathway in skeletal muscles and AdipoR2 that activates peroxisome proliferator-activated receptors (PPARα) pathway in the liver to increase insulin sensitivity and decrease inflammation. Experimental evidence suggests that AdipoR1 and R2 serve as major AdipoRs in* in vivo* conditions (Yamauchi et al., 2007). The prevalence of obesity has increased sharply in the recent times. Adiponectin levels are negatively correlated with body mass but positively correlated with reduction in body weight. HMW adiponectin is decreased in obesity and type II diabetes and increased in type I diabetes (Yang et al., 2001; Pajvani and Scherer, 2003; Yatagai et al., 2003; Flier, 2004; Wolf et al., 2004; Pereira et al., 2012). It is produced in large quantities by normal fat cells and less by fat cells (Nawrocki et al., 2006). High levels of HMW adiponectin and total adiponectin was observed in children suffering with Prader–Willi syndrome despite of profound obesity and hypoinsulinaemia (Haqq et al., 2007). HMW adiponectin levels are also decreased during gestational diabetes (Retnakaran et al., 2007). *In vitro* treatment of adipocytes with pioglitazone, an antidiabetic drug, increased HMW adiponectin levels (Bodles et al., 2006). AdipoRon, a small synthetic molecule, acts as agonist of adiponectin. When administered orally in mice, it delivered the same effects of adiponectin in muscle and liver cells and alleviated insulin resistance and type-II diabetes (Okada-Iwabu et al., 2013). Therefore, HMW adiponectin or its agonist can act as novel therapeutic strategy as a treatment to counteract these diseases as suggested by Yamauchi and Kadowaki (2008).

Obesity also results in the development of several other diseases like diabetes, cancer, fatty liver, and cardiovascular disorders which are positively related to angiogenesis. In experimental mice, adiponectin inhibits endothelial cell proliferation and migration, primary tumor growth, and reduces atherosclerosis. Adiponectin alleviates alcoholic and obese induced fatty liver diseases (Xu et al., 2003), acts as anti-inflammatory hormone in the repair of liver injury induced by CCl_4_ (Yoda-Murakami et al., 2001), and suppresses liver fibrosis (Kamada et al., 2003). Adiponectin plays an important role in energy homeostasis too (Yamauchi et al., 2003; Qi et al., 2004). Hypoadiponectin results in twofold increase in coronary artery diseases in men (Kumada et al., 2003), but development of atherosclerosis was suppressed in mice with increased plasma levels of adiponectin (Okamoto et al., 2002; Trivedi et al., 2012a). It was shown that adiponectin induces antiangiogenesis and antitumor activity *via* caspase-mediated endothelial cell apoptosis (Brakenhielm et al., 2004). Adiponectin shows anti-inflammatory role in murine colitis also (Arsenescu et al., 2011). Adiponectin can prevent fetal alcohol syndrome by protecting hippocampal neurons against alcohol induced apoptosis (Naseer et al., 2014). Adiponectin alleviates ceramidase activity, helps in the reduction of palmitate-induced cell death and over production of adiponectin decreases caspase-8-mediated death (Holland et al., 2011).

## Osmotin Mimics Adiponectin

Osmotin, a naturally occurring plant protein mimics human adiponectin. Osmotin shares structural and functional homology with adiponectin and not sequence similarity (Min et al., 2004). Osmotin exerts its action by binding to a seven-transmembrane-domain receptor-like protein encoded by *PHO36*, while a mammalian homolog of PHO36 receptor is the human hormone AdipoR1. Domain I (core protein) of osmotin resembles the structural homolog of β-barrel domain of adiponectin. Like adiponectin, osmotin binds to AdipoRs and induces AMP kinase phosphorylation in mammalian C2C12 myocytes (Narasimhan et al., 2005). Osmotin activity was studied on *in vitro* and animal models. When administered intravenously in experimental rats, it acts as adiponectin agonist in obesity and type-II diabetes, and also displayed antiatherosclerotic activity (Trivedi et al., 2012a,b). Like adiponectin, osmotin also exhibited similar functional activity in *in vitro* cultured human synovial fibroblasts (Miele et al., 2011). Like adiponectin, osmotin exerted similar anti-inflammatory function in murine colitis (Arsenescu et al., 2011). Subcutaneous administration of osmotin, protects rat pups from ethanol induced apoptosis in cortical and hippocampal neurons (Naseer et al., 2014). Osmotin is resistant to pepsin digestion and heat treatment, and shows significant IgE binding and cross reacts with tomato and apple allergens. Allergenicity of osmotin can be reduced by mutations in IgE binding epitopes (Sharma et al., 2011, 2013a). Furthermore, *in silico* analysis suggests that both osmotin and adiponectin interact with the same drugs.

## Conclusion

Osmotin, a multifaceted plant protein confers tolerance to both biotic and abiotic stresses. Adiponectin, an antidiabetic and antiatherosclerotic protein is reduced in obese patients and leads to several diseases including coronary artery disease, inflammation, and liver diseases. Osmotin shows homology with human hormone adiponectin given that osmotin not only induces AMP kinase phosphorylation in mammalian C2C12 myocytes *via* AdipoRs, but also binds to the AdipoR1 by activating the same signaling path of adiponectin. Osmotin and adiponectin involve in antitumor activity by inhibiting p^53^ and suppressing caspase activity. *In vitro* and animal model studies suggest that, like AdipoRon and pioglitazone, osmotin acts as agonist for adiponectin. Due to the multiple activities of osmotin, it can be explored as an attractive option as agonist for adiponectin in treating adiponectin deficiency diseases in humans besides its function in biotic and abiotic stress tolerance in crop plants.

## Author Contributions

All the authors of the manuscript meet the essential criteria of the publication.

## Conflict of Interest Statement

The authors declare that the research was conducted in the absence of any commercial or financial relationships that could be construed as a potential conflict of interest.
